# Abdominal Tremor in Idiopathic Parkinson’s Disease: A Case Report

**DOI:** 10.5334/tohm.822

**Published:** 2024-01-12

**Authors:** Raumin S. Neuville, Anna E. Morenkova

**Affiliations:** 1 School of Medicine, University of California, Irvine, Irvine, 10 CA 92617, USA; 2 Department of Neurology, University of California, Irvine, S. Manchester Ave., Ste 206, Orange, CA 92868, USA

**Keywords:** Tremor, Parkinson’s disease (PD), Abdominal tremor, Levodopa-responsive

## Abstract

**Background:**

Tremor in Parkinson’s disease (PD) is commonly seen in the upper extremities and can involve the lower extremities and mouth. We present a case of a patient with idiopathic PD who presented with abdominal tremor.

**Case Report:**

A 40-year-old man with a 2-year history of subjective weakness and stiffness in the right arm and leg, followed by emergence of a right hand tremor, subsequently developed abdominal tremor. Patient experienced marked improvement of both abdominal and hand tremor and mobility of the right limbs with levodopa.

**Discussion:**

Our case report serves as the second only published report of abdominal tremor in an idiopathic PD patient.

**Highlights:**

Tremor in Parkinson’s disease (PD) commonly affects the upper and lower extremities and mouth. We describe a 40-year-old man with PD who developed abdominal tremor which was brought under control with levodopa. This case is one of only two published reports of abdominal tremor in PD.

## Introduction

Tremor, particularly at rest, is one of the cardinal symptoms of Parkinson’s disease (PD). It has been demonstrated to be present in up to 80% of patients with postmortem-confirmed PD [1]. Resting tremor is a repetitive, low frequency movement that commonly occurs in the upper extremities and can also affect the legs and mouth. There has been only a single documented case in the literature reporting tremor involving the abdominal muscles of an individual with PD [2]. Other documented cases of abdominal tremor have been associated with thyrotoxicosis and orthostatic tremor [3,4,5]. We present a case of a male with idiopathic Parkinson’s disease with severe bilateral asymmetrical bradykinesia and rigidity, mild unilateral resting tremor of hand and the very rare occurrence of tremor affecting the abdominal wall. We include a video of this individual’s abdominal tremor in a sitting position.

## Case Description

A 40-year-old man who works as a construction worker presented with at least 2 years of subjective weakness and stiffness of the right arm and leg, and subsequently 1 year later, mild tremor of the right hand. The tremor later progressed to affect his abdomen and was most intense in a sitting and standing position, less prominent when supine, and not observed when ambulating. He reported that the abdominal tremor would worsen when he was using his right arm and with anxiety. His medications included lidocaine patch and cyclobenzaprine, prescribed elsewhere for the treatment of his lower back pain. There was no history of exposure to neuroleptics and other antidopaminergic medications, lithium, or valproate, no toxic exposures, and his thyroid function was normal. He had no family history of PD or other neurologic conditions. Physical exam demonstrated visible moderate frequency, mild-to-moderate amplitude rhythmic contractions of the rectus abdominis muscles which markedly increased in amplitude during distraction (Video 1). The tremor appears to be bilateral and more pronounced on the right, although the possibility remains of a unilateral abdominal tremor with oscillations transferred to the contralateral rectus abdominis.

**Video 1:** **Video shows clinical features of the patient with abdominal tremor.** Showing patient in seated position with continuous, rhythmic contractions of the rectus abdominis muscles (right greater than left) that is mild to moderate in amplitude. (Written consent was obtained from the patient in this video to be submitted/published in this journal).

Additional findings included severe asymmetrical bradykinesia and rigidity in the upper and lower extremities that was more pronounced on the right, and moderate frequency, low amplitude intermittent rest and re-emergent postural tremor of the right hand, independent from the abdominal tremor. Workup for secondary causes of parkinsonism was negative. 1.5 Tesla Magnetic Resonance Imaging (MRI) of the brain without gadolinium contrast was normal. DaT (Dopamine Transporter) scan was not pursued since the clinical presentation and examination findings were consistent with severe parkinsonism with asymmetric motor findings, favoring primary parkinsonism, and the workup for secondary causes of parkinsonism was negative, including no history of exposure to antidopaminergic medications and normal brain MRI. The UK Parkinson’s Disease Society Brain Bank Clinical Diagnostic Criteria were used to establish diagnosis of PD in this patient. He was started on carbidopa-levodopa with gradual titration up to 600 mg a day and, at 10-week follow-up, neurological examination demonstrated absence of both abdominal and hand tremor. The patient reported alleviation of tremor lasting for 5 hours following every carbidopa-levodopa dose and improvement of general mobility and stiffness. The improvement of his tremor was sustained and there was continued improvement of his severe parkinsonism upon further increase of levodopa regimen to 1200 mg a day. Unified Parkinson’s Disease Rating Scale (UPDRS) Part III was 50.0 at initial visit and 26.0 10 weeks after the initiation of levodopa. After 11 months of levodopa treatment, neurologic examination in the ON state 4 hours after levodopa dose demonstrated sustained control of abdominal tremor and UPDRS Part III score of 7.0 (Video 2).

**Video 2:** **Video demonstrates absence of abdominal tremor at 11 months since initiation of levodopa treatment.** Showing patient in seated position with no observable abdominal tremor at rest or when the patient was distracted by conversation and formal motor examination.

## Discussion

This case represents the rare presentation of abdominal tremor in an individual with PD. Only a single case report has been published that showcased a 66-year-old male with PD and abdominal tremor at rest in supine position, which improved with a daily regimen of pramipexole [2]. The characteristic rhythmic, symmetrical contractions and exacerbation of abdominal tremor by distraction present in this case of abdominal tremor are characteristic features of parkinsonian tremor and make a functional movement disorder less likely. The improvement from levodopa supports the parkinsonian nature of the tremor and underlying dopaminergic dysfunction. Of interest is the possible bilateral abdominal rectus involvement whereas appendicular tremor was unilateral.

Rest tremor is usually low frequency and involves the hands and lower extremities. The underlying pathophysiology of tremor in PD is not well understood. It has been suggested that tremor is linked to two circuits, the basal ganglia and cerebello-thalamo-cortical circuit that triggers the tremor episode and produces the tremor, respectively [6]. The response of tremor to levodopa can be variable for those with PD and tremor can become resistant to medication as the disease progresses [7]. Additionally, cognitive stress can not only increase the intensity of the parkinsonian tremor, as seen in this case, but can also reduce the therapeutic effect of levodopa [8]. Our case report serves as the second only published report of abdominal tremor in an individual with PD.

## Data Accessibility Statement

All of the case report information is part of the patient’s confidential medical record. The case report video can be made public. Requests regarding the data in this report should be addressed to the authors.

## References

[1] Gelb DJ, Oliver E, Gilman S. Diagnostic criteria for Parkinson disease. Arch Neurol. 1999; 56(1): 33–39. DOI: 10.1001/archneur.56.1.33 9923759

[2] Tanaka N, Hayashi T, Tsuji S, Iwata A. Abdominal Tremor in Parkinson’s Disease. Mov Disord Clin Pract. 2015; 2(1): 104–105. DOI: 10.1002/mdc3.12119 30713888 PMC6353404

[3] Mousele C, Bentley P, Tai YF. A Rare Presentation of Orthostatic Tremor as Abdominal Tremor. Tremor Other Hyperkinet Mov (N Y). 2018; 8: 603. DOI: 10.5334/tohm.423 30622838 PMC6315046

[4] Lenka A, Jankovic J. Tremor Syndromes: An Updated Review. Frontiers in Neurology. 2021; 12. DOI: 10.3389/fneur.2021.684835 PMC8350038 34381412

[5] Kelly DM, Lynch T, Casserly LF. Abdominal tremor in thyrotoxicosis. Neurology. 2017; 89(13): 1424–1425. DOI: 10.1212/WNL.0000000000004403 28821688 PMC10681063

[6] Helmich RC, Hallett M, Deuschl G, Toni I, Bloem BR. Cerebral causes and consequences of parkinsonian resting tremor: a tale of two circuits? Brain. 2012; 135(Pt 11): 3206–3226. DOI: 10.1093/brain/aws023 22382359 PMC3501966

[7] Oertel W, Fahn S. Parkinsonism. In: Neurological Disorders: Course and Treatment. Brandt T, Caplan LR, Dichgans J, Diener HC, Kennard C (eds.). Gulf Professional Publishing; 2003.

[8] Zach H, Dirkx MF, Pasman JW, Bloem BR, Helmich RC. Cognitive Stress Reduces the Effect of Levodopa on Parkinson’s Resting Tremor. CNS Neurosci Ther. 2017; 23(3): 209–215. DOI: 10.1111/cns.12670 28071873 PMC5324662

